# Spire-Type Actin Nucleators Cooperate with Formin-2 to Drive Asymmetric Oocyte Division

**DOI:** 10.1016/j.cub.2011.04.029

**Published:** 2011-06-07

**Authors:** Sybille Pfender, Vitaliy Kuznetsov, Sandra Pleiser, Eugen Kerkhoff, Melina Schuh

**Affiliations:** 1 Medical Research Council, Laboratory of Molecular Biology, Cambridge CB2 0QH, United Kingdom; 2 Molecular Cell Biology Laboratory, Bavarian Genome Research Network, Department of Neurology, University Hospital Regensburg, Franz-Josef-Strauß-Allee 11, D-93053 Regensburg, Germany

## Abstract

Oocytes mature into eggs by extruding half of their chromosomes in a small cell termed the polar body. Asymmetric oocyte division is essential for fertility [1], but despite its importance, little is known about its mechanism. In mammals, the meiotic spindle initially forms close to the center of the oocyte. Thus, two steps are required for asymmetric meiotic division: first, asymmetric spindle positioning and second, polar body extrusion. Here, we identify Spire1 and Spire2 as new key factors in asymmetric division of mouse oocytes. Spire proteins are novel types of actin nucleators that drive nucleation of actin filaments with their four WH2 actin-binding domains [2–6]. We show that Spire1 and Spire2 first mediate asymmetric spindle positioning by assembling an actin network that serves as a substrate for spindle movement. Second, they drive polar body extrusion by promoting assembly of the cleavage furrow. Our data suggest that Spire1 and Spire2 cooperate with Formin-2 (Fmn2) to nucleate actin filaments in mouse oocytes and that both types of nucleators act as a functional unit. This study not only reveals how Spire1 and Spire2 drive two critical steps of asymmetric oocyte division, but it also uncovers the first physiological function of Spire-type actin nucleators in vertebrates.

## Graphical abstract

**Graphical Abstract:**

**Figure:**
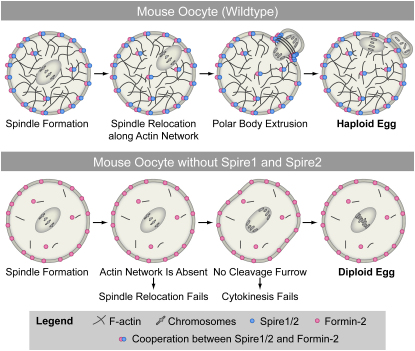

**Highlights:**

► Spire1 and Spire2 mediate asymmetric spindle positioning in mouse oocytes ► They assemble an actin network that serves as a substrate for spindle movement ► Spire1 and Spire2 drive polar body extrusion by promoting cleavage furrow assembly ► Spire1 and Spire2 cooperate with Formin-2 in a functional unit

## Results and Discussion

### Spire1 and Spire2 Mediate Asymmetric Spindle Positioning by Assembling a Cytoplasmic Actin Network

We first tested whether the mammalian Spire genes, *Spire1* and *Spire2*, are expressed in mouse oocytes. Quantitative real-time PCR revealed that both *Spire1* and *Spire2* are expressed and that mRNA levels in oocytes are significantly higher than in other tissues (Figures S1A and S1B).

To investigate whether one of these proteins is required for asymmetric oocyte division, we first depleted *Spire1* and *Spire2* individually by RNAi. Neither individual depletion of *Spire1* nor individual depletion of *Spire2* blocked asymmetric spindle positioning, actin network assembly, or polar body extrusion (Figures S1D and S1E and S1G–S1L). Thus, if individually depleted, *Spire1* and *Spire2* are dispensable for asymmetric oocyte division.

**Figure 1:**
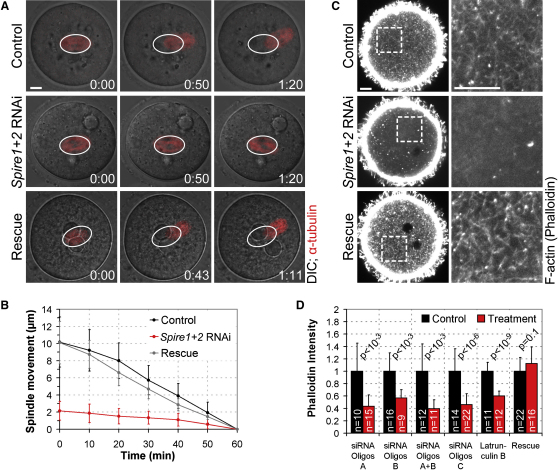
Spire1 and Spire2 Mediate Asymmetric Spindle Positioning by Assembling a Cytoplasmic Actin Network (A) Spindle movements in live oocytes expressing mCherry-α-tubulin (red, microtubules, merged with differential interference contrast [DIC]). Oocytes were injected with control siRNA (Control), *Spire1* and *Spire2* siRNAs (*Spire1+2* RNAi) and *Spire1* and *Spire2* siRNAs together with human *Spire1* and *Spire2* mRNAs (rescue). White ovals mark initial spindle positions. Time is given in hr:min. The scale bar represents 10 μm. See also Movie S1. (B) The spindle was tracked in oocytes in 3D data sets (13 sections, every 7 μm) as shown in (A). Spindle movements in control (black; v = 0.20 ± 0.06 μm/min; n = 13), *Spire1*+*2* RNAi (red; v = 0.03 ± 0.03 μm/min; n = 23; p < 10^−12^), and rescue experiments (gray; v = 0.16 ± 0.04 μm/min; n = 9; p = 0.09) are plotted. (C) Oocytes from control, *Spire1+2* RNAi, and rescue experiments were fixed during spindle relocation and stained with Alexa Fluor 488 phalloidin so that F-actin would be labeled. Boxed regions are magnified on the right. Scale bars represent 10 μm. (D) The mean intensity of the cytoplasmic phalloidin staining was measured in oocytes (as shown in [C]) that were injected with control siRNA (black); with different oligo sets of *Spire1* and *Spire2* siRNAs (red); with *Spire1* and *Spire2* siRNAs together with human *Spire1* and *Spire2* mRNAs (rescue; red); and in oocytes treated with 4 μM Latrunculin B or DMSO (Latrunculin B (red); DMSO (black)). Data are means ± SD. p values were calculated with a Student's t test.

Because Spire1 and Spire2 have highly similar protein structures [7], we hypothesized that they might act redundantly, and we tested this hypothesis by codepleting both proteins. First, we analyzed whether Spire1 and Spire2 are required for asymmetric spindle positioning. Confocal imaging of live oocytes revealed that asymmetric spindle positioning failed if *Spire1* and *Spire2* were codepleted (Figure 1; see also Figure S1F and Movie S1). Automated 3D tracking of the spindle confirmed that the spindle moved significantly more slowly than in control oocytes injected with scrambled negative control siRNA (Figure 1B; see Supplemental Information for all quantification methods). Asymmetric spindle positioning could be rescued by injection of mRNAs that encode human Spire1 and Spire2 and are resistant to the siRNAs that target the mouse mRNAs (Figures 1A and 1B), and therefore failure of asymmetric spindle positioning was not due to a nonspecific RNAi response. Confirming the redundancy of Spire1 and Spire2, individual expression of human Spire1 or Spire2 also rescued asymmetric spindle positioning (Figures S2A and S2B).

We next investigated the mechanism by which the Spire proteins mediate asymmetric spindle positioning. In oocytes from humans and all other mammals analyzed so far, asymmetric spindle positioning depends on F-actin [8]. In previous studies, we and others identified a cytoplasmic actin network that is required for asymmetric spindle positioning [9, 10]. The actin network fills the oocyte's cytoplasm and provides the substrate for myosin-dependent spindle movement [9, 11]. To address whether Spire1- and Spire2-dependent F-actin nucleation is required for assembly of the actin network, we analyzed the network in the center of the oocyte by high-resolution confocal microscopy. We found that the actin network was greatly reduced if *Spire1* and *Spire2* were codepleted, down to background levels similar to those seen in oocytes treated with the F-actin-depolymerising drug Latrunculin B (Figures 1C and 1D). The fact that we could rescue the network by expressing human Spire1 and Spire2 (Figures 1C and 1D) indicated that the absence of the actin network was specifically due to depletion of *Spire1* and *Spire2*. Other actin structures, such as cortical F-actin, were intact (Figure 1C). We conclude that Spire1 and Spire2 mediate asymmetric spindle positioning by assembling the cytoplasmic actin network.

### Spire1 and Spire2 Drive Polar Body Extrusion by Promoting Assembly of the Cleavage Furrow

**Figure 2:**
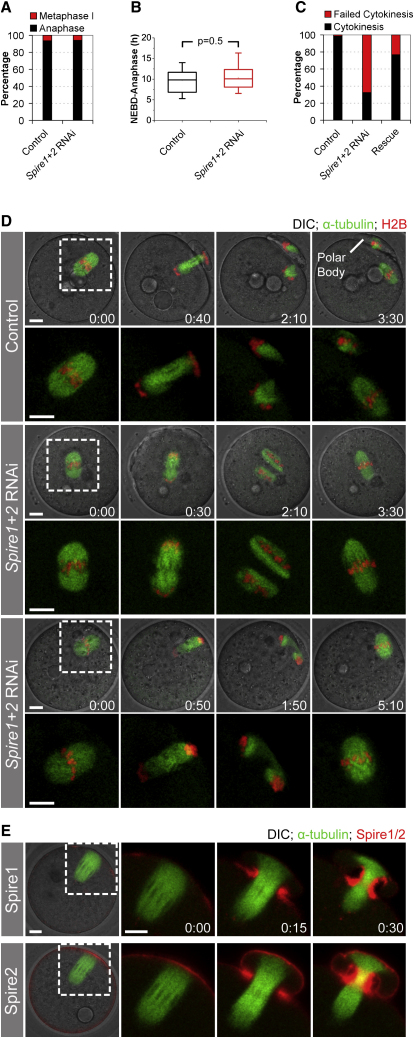
Spire1 and Spire2 Drive Polar Body Extrusion by Promoting Assembly of the Cleavage Furrow (A) Live oocytes injected with control siRNA (control; n = 61) or *Spire1* and *Spire2* siRNAs (*Spire1+2* RNAi; n = 96) were monitored by long-term time-lapse microscopy, and arrest in metaphase I (red) or progression into anaphase (black) were scored. (B) The time between nuclear-envelope breakdown (NEBD) and anaphase onset was measured in live control oocytes (n = 30) and *Spire1+2* RNAi-treated oocytes (n = 33). p values were calculated with a Student's t test. (C) Live oocytes injected with control siRNA (control; n = 57), *Spire1* and *Spire2* siRNAs (*Spire1+2* RNAi; n = 90), and *Spire1* and *Spire2* siRNAs together with human *Spire1* and *Spire2* mRNAs (rescue; n = 30), were scored for successful cytokinesis (cytokinesis) or cytokinetic failure upon anaphase onset (failed cytokinesis). (D) Anaphase in live control and *Spire1+2* RNAi-treated oocytes expressing EGFP-α-tubulin (green, microtubules) and H2B-mRFP (red, chromosomes) merged with DIC. Boxed regions are magnified without DIC below. Scale bars represent 10 μm. Time is given in hr:min. See also Movie S2. (E) Polar body extrusion in live oocytes expressing EGFP-α-tubulin (green, microtubules) and Spire1-mCherry (upper row) or Spire2-mCherry (lower row). The first image is merged with DIC. Time-lapse images of boxed regions without DIC are shown on the right. Scale bars represent 10 μm. Time is given in hr:min. See also Movie S3.

We next asked whether Spire1 and Spire2 are also required for polar body extrusion. We found that only 30% (n = 96) of *Spire1* and *Spire2* codepleted oocytes extruded a polar body, whereas 92% (n = 61) of control oocytes did so. Polar body extrusion could fail because meiosis does not progress into anaphase if asymmetric spindle positioning is abolished, or it could fail because Spire1 and Spire2 are directly involved in polar body formation. Long-term time-lapse imaging of live oocytes revealed that meiotic progression was not affected; neither the efficiency of progression into anaphase (Figure 2A) nor the timing of anaphase onset (Figure 2B) was significantly different from that in control oocytes. Instead, 68% (n = 90) of *Spire1* and *Spire2* codepleted oocytes failed to assemble a cleavage furrow upon anaphase onset (Figures 2C and 2D). After telophase, two separate spindles formed and finally fused with each other to create a joined metaphase II spindle, resulting in a diploid instead of a haploid egg (Figure 2D; Movie S2). Cytokinetic failure was specifically due to depletion of *Spire1* and *Spire2*, as shown by the fact that we could rescue cytokinesis by expressing human Spire1 and Spire2 (Figure 2C; Figures S2C and S2D). The large distance between the spindle and the cortex was not the cause of failed furrow ingression: first, cytokinesis also failed if the spindle formed close to the cortex in oocytes that had an eccentric nucleus (Figure 2D, row 3; Movie S2), and second, oocytes did divide symmetrically if the actin network density was artificially increased so that asymmetric spindle positioning was blocked (Figure S3; details in legend). Together, these data show that Spire1 and Spire2 are required for assembly of the cleavage furrow during polar body extrusion. One obvious model for the function of Spire1 and Spire2 in furrow assembly is that they are required for actin organization in the contractile ring. Consistent with this model, we found that fluorescent fusion proteins of both Spire1 and Spire2 are strongly enriched in the cleavage furrow during polar body formation (Figure 2E; Movie S3).

### Spire1 and Spire2 Cooperate with Fmn2 in a Functional Unit

**Figure 3:**
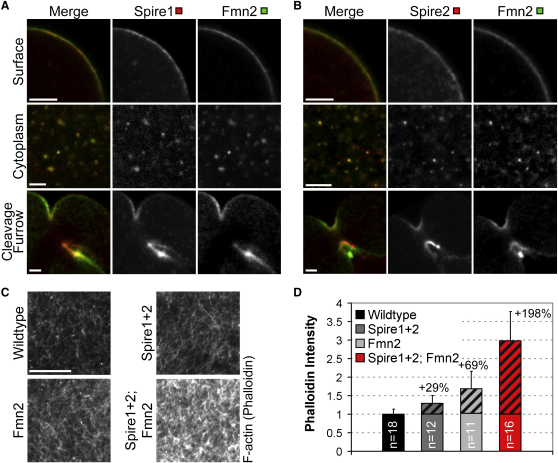
Spire1 and Spire2 Cooperate Synergistically with Fmn2 to Assemble F-Actin in Mouse Oocytes (A and B) Colocalization of Spire1-mCherry (A) or Spire2-mCherry (B) (red) and Fmn2-mEGFP (green) at the oocyte surface (upper row), in the cytoplasm (middle row), and in the cleavage furrow (lower row). (C) Wild-type oocytes (wild-type) and oocytes overexpressing Fmn2-mCherry (Fmn2), Spire1-mCherry and Spire2-mCherry (Spire1+2), or Spire1-mCherry and Spire2-mCherry together with Fmn2-mCherry (Spire1+2; Fmn2) were fixed during spindle relocation and stained with Alexa Fluor 488 phalloidin to label F-actin. Scale bars represent 10 μm. (D) The mean intensity of the cytoplasmic actin network was measured in oocytes as shown in (C). Percentages specify the increase of the network intensity in comparison to that of controls (highlighted by stripes).

The phenotypes of oocytes that lack Spire1 and Spire2 are remarkably similar to those of oocytes that lack the formin Fmn2. Without Fmn2, asymmetric spindle positioning fails because the cytoplasmic actin network is absent [1, 9, 10], and polar body extrusion fails because of cytokinetic defects [12]. Moreover, the expression profiles of *Spire1* and *Spire2* are very similar to that of *Fmn2* (Figures S1A–S1C). In the FMN subfamily of formins, to which Fmn2 belongs, a conserved sequence motif that can mediate interaction with Spire proteins in vitro was recently identified [13, 14]. Given that both types of actin nucleators interact in vitro and that their depletion results in identical phenotypes, it seemed likely that Spire1 and Spire2 cooperate with Fmn2 in a functional unit to drive asymmetric spindle positioning and polar body extrusion. To test this hypothesis, we first analyzed whether Spire1 and Spire2 colocalize with Fmn2 in mouse oocytes. Indeed, we found that both nucleators colocalize at the oocyte surface, in the cytoplasm, and in the cleavage furrow, as judged by localization of fluorescent fusion proteins (Figures 3A and 3B). We next used an overexpression assay to investigate whether both nucleators cooperate to assemble the cytoplasmic actin network. We measured the increase of the density of the actin network in oocytes that overexpress either Spire1 and Spire2 alone or Fmn2 alone and compared it to that in oocytes that overexpress both types of nucleators at the same time (Figures 3C and 3D). If Spire1 and Spire2 cooperate with Fmn2, then co-overexpression should cause a larger increase than individual overexpression of both types of nucleators together. Indeed, we found that co-overexpression caused an increase of 198%, whereas overexpression of Spire1 and Spire2 or of Fmn2 alone caused an increase of only 29% or 69%, respectively (Figure 3D). These results suggest that Spire1 and Spire2 interact with Fmn2 in a cooperative manner to assemble F-actin in mouse oocytes.

**Figure 4:**
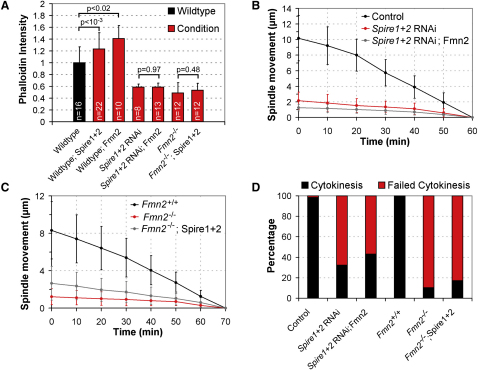
Spire1 and Spire2 Cooperate Interdependently with Fmn2 in a Functional Unit (A) The mean intensity of the cytoplasmic phalloidin staining was measured in wild-type oocytes (black column) and in wild-type oocytes overexpressing Spire1-mCherry and Spire2-mCherry (wild-type; Spire1+2) or Fmn2-mCherry (wild-type; Fmn2); in *Spire1-* and *Spire2*-depleted oocytes without (*Spire1+2* RNAi) or with (*Spire1+2* RNAi; Fmn2) Fmn2-mCherry; in oocytes from *Fmn2* knockout mice without (*Fmn2^−/−^*) or with (*Fmn2^−/−^*; Spire1+2) Spire1-mCherry and Spire2-mCherry (red columns). Data are means ± SD. p values were calculated with a Student's t test. (B) The spindle was tracked in oocytes injected with control siRNA (black; v = 0.20 ± 0.06 μm/min; n = 13), in *Spire1-* and *Spire2*-depleted oocytes without (red; v = 0.03 ± 0.03 μm/min; n = 23; p < 10^−12^) or with (gray; v = 0.02 ± 0.01 μm/min; n = 11; p < 10^−8^) Fmn2-mCherry. (C) The spindle was tracked in wild-type oocytes (black; 0.11 ± 0.05 μm/min; n = 23); in oocytes from *Fmn2^−/−^* mice without (red; 0.02 ± 0.01 μm/min; n = 12; p < 10^−7^) or with (gray; 0.02 ± 0.01 μm/min; n = 12; p < 10^−8^) Spire1-mCherry and Spire2-mCherry. (D) Successful cytokinesis (cytokinesis) or failure of cytokinesis (failed cytokinesis) were scored for oocytes injected with negative control siRNA (control; n = 57), for *Spire1-* and *Spire2*-depleted oocytes without (*Spire1+2* RNAi; n = 90) or with Fmn2-mCherry (*Spire1+2* RNAi; Fmn2; n = 14); for oocytes from *Fmn2^+/+^* mice (*Fmn2^+/+^* n = 31), Fmn2*^−/−^* mice without (*Fmn2^−/−^* n = 30) or with (*Fmn2^−/−^*; Spire1+2 n = 41) Spire1-mCherry and Spire2-mCherry.

Next, we investigated whether Spire1 and Spire2 cooperate with Fmn2 in a functional unit in which both interaction partners depend on each other to fulfil their function. We first tested whether both nucleators depend on each other to assemble F-actin. When we overexpressed either Fmn2 or both Spire1 and Spire2 in wild-type oocytes in which the other type of nucleator was present, both caused a significant increase in the density of the actin network (Figure 4A). We then tested whether a similar increase is observed if the other nucleator is absent. We found that neither overexpression of Fmn2 in *Spire1* and *Spire2* co-depleted oocytes nor overexpression of Spire1 and Spire2 in oocytes from *Fmn2^−/−^* mice increased the network density (Figure 4A), indicating that both actin nucleators depend on each other to assemble F-actin in mouse oocytes. Consistently, neither overexpression of Fmn2 in *Spire1* and *Spire2* codepleted oocytes, nor overexpression of Spire1 and Spire2 in oocytes from *Fmn2^−/−^* mice rescued asymmetric spindle positioning (Figures 4B and 4C) or polar body extrusion (Figure 4D). Together, our data suggest that Spire1 and Spire2 cooperate with Fmn2 in a functional unit in which both types of nucleators depend on each other.

In contrast to interaction between the Spire proteins and Fmn2, interaction with members of the Rho family of small GTPases as reported for other types of actin nucleators was dispensable for the activation of actin assembly. Neither dominant-negative variants nor peptide inhibitors of Rho GTPases blocked the assembly of the cytoplasmic actin network (Figures S4A and S4B). Instead, we found that the expression level of *Spire1, Spire2*, and *Fmn2* determined the actin network density (Figures S4C and S4D).

In summary, this study identifies Spire1 and Spire2 as new essential factors in asymmetric oocyte division. Spire1 and Spire2 cooperate with Fmn2 to assemble the cytoplasmic actin network that mediates asymmetric spindle positioning and to drive polar body extrusion by promoting assembly of the cleavage furrow. The mechanism of asymmetric oocyte division is likely to be conserved between humans and mice; human and mouse oocytes share many features, including an F-actin-dependent mechanism of asymmetric spindle positioning [15]. Also, the function of Spire1, Spire2, and Fmn2 is likely to be conserved given that recently published microarray data indicate that all three genes are highly expressed in human oocytes [16]. We show that depletion of *Spire1* and *Spire2* results in diploid instead of haploid eggs; likewise, fertilization of oocytes from *Fmn2* knockout mice was shown to result in polyploid embryos and pregnancy loss [1]. Therefore, it will be important to investigate whether defects in the *SPIRE1*, *SPIRE2*, and *FMN2* genes contribute to cases of pregnancy loss and unexplained infertility in humans [17].

## Experimental Procedures

### Preparation and Culture of Oocytes

All mice were maintained in a specific pathogen-free environment according to UK Home Office regulations. Oocytes were isolated from ovaries of 8-week-old FVB, 129/Sv, or *Fmn2^−/−^* mice [1], cultured, and microinjected as described in detail before [18]. siRNAs were purchased from QIAGEN. siRNA sequences are listed in the Supplemental Information. siRNAs were injected to a final concentration of 5.5 nM into follicle-enclosed oocytes from 10- to 11-day-old (C57BL × CBA) F1 females and then cultured for 10 days [19, 20]. In some experiments, oocytes were microinjected with 60 μg/ml Exoenzyme C3 (Calbiochem; final concentration in oocytes: 120 nM) or bovine serum albumin (BSA, from Sigma) or treated with 3 μg/ml Toxin B (Calbiochem), 4 μM Latrunculin B (Calbiochem), or corresponding amounts of dimethyl sulfoxide (DMSO, from Sigma).

### Expression Constructs and mRNA Synthesis

To generate the constructs for in vitro mRNA synthesis, we fused the previously published protein coding sequences with EGFP (Clontech) or mCherry [21] to obtain Spire1-EGFP and Spire1-mCherry [22], Spire2-EGFP and Spire2-mCherry [13], mEGFP-α-tubulin and mCherry-α-tubulin [23], and mCherry-Cdc42(T17N) [24] and mCherry-Rac1(T17N) [25] and inserted these into pGEMHE [26] for in vitro transcription. These constructs as well as pGEMHE-H2B-mRFP1 and pGEMHE-EGFP-MAP4 [18] were linearized with AscI. Capped mRNA was synthesized with T7 polymerase (mMessage mMachine kit, according to the instructions of the manufacturer [Ambion]) and dissolved in 11 μl water. pCS2-Fmn2-EGFP [1] and pCS2-Fmn2-mCherry [9] were linearized with KpnI or BssHII, respectively. Capped mRNAs were synthesized with SP6 polymerase (mMessage mMachine kit, Ambion), polyadenylated [Poly(A) Tailing Kit, Ambion], and dissolved in 6 μl water. mRNA concentrations were determined on ethidium bromide agarose gels by comparison with an RNA standard (Ambion).

### Confocal Microscopy

Images were acquired with a Zeiss LSM710 confocal microscope equipped with a Zeiss environmental incubator box, with a 40× C-Apochromat 1.2 NA water immersion objective lens for live oocytes and a 63× C-Apochromat 1.2 NA water immersion objective lens for fixed oocytes, as previously described [18]. In some images, shot noise was reduced with a Gaussian filter.

### Phalloidin Staining

Oocytes were fixed for 30–60 min at 37°C in 100 mM HEPES (pH 7) (titrated with KOH), 50 mM ethylene glycol tetra-acetic acid (EGTA) (pH 7) (titrated with KOH), 10 mM MgSO_4_, 2% formaldehyde (MeOH free) and 0.2% Triton X-100, on the basis of previously published methods [27]. Oocytes were left in phosphate-buffered saline (PBS) and 0.1% Triton X-100 overnight at 4°C. F-actin was stained for 2 hr with Alexa Fluor 488 phalloidin (Molecular Probes; 0.33 μM /10 units/ml) in PBS, 0.1% Triton X-100, and 3% BSA.

## Supplemental Information

Document S1. Supplemental Experimental Procedures and Four Figures

Movie S1. Spire1 and Spire2 Drive Asymmetric Spindle Positioning. Related to Figure 1 Time-lapse imaging (one image every 10 min) during asymmetric spindle positioning of oocytes expressing mEGFP-α-tubulin (green, microtubules) and H2B-mRFP (red, chromosomes). Oocytes were injected with scrambled negative control siRNA (left) and *Spire1* and *Spire2* siRNAs (right).

Movie S2. Spire1 and Spire2 Drive Polar Body Extrusion. Related to Figure 2 Time-lapse imaging (one image every 10 min) during anaphase of oocytes expressing mEGFP-α-tubulin (green, microtubules) and H2B-mRFP (red, chromosomes). Oocytes were injected with scrambled negative control siRNA (left) and *Spire1* and *Spire2* siRNAs (middle and right). Same oocytes as in Figure 2D.

Movie S3. Spire1 and Spire2 Localize to the Cleavage Furrow during Polar Body Extrusion. Related to Figure 2 Time-lapse imaging (one image every 5 min) during anaphase in ooocytes overexpressing mEGFP−α-tubulin (green, microtubules) and Spire1-mCherry (red; left) or Spire2-mCherry (red; right). Same oocytes as in Figure 2E.
