# Supplementary material for: Spire-Type Actin Nucleators Cooperate with Formin-2 to Drive Asymmetric Oocyte Division
Source: Curr Biol. 2011 Jun 7;21(11):955–60. doi: 10.1016/j.cub.2011.04.029 (PMC3128265; doi:10.1016/j.cub.2011.04.029)
Supplement: Document S1. Supplemental Experimental Procedures and Four Figures [file mmc1.pdf]

Current Biology Volume *21*

Supplemental Information

Spire-Type Actin Nucleators Cooperate with Formin-2 to Drive Asymmetric Oocyte Division

Sybille Pfender, Vitaliy Kuznetsov, Sandra Pleiser, Eugen Kerkhoff, and Melina Schuh

Pfender et al. Figure S1

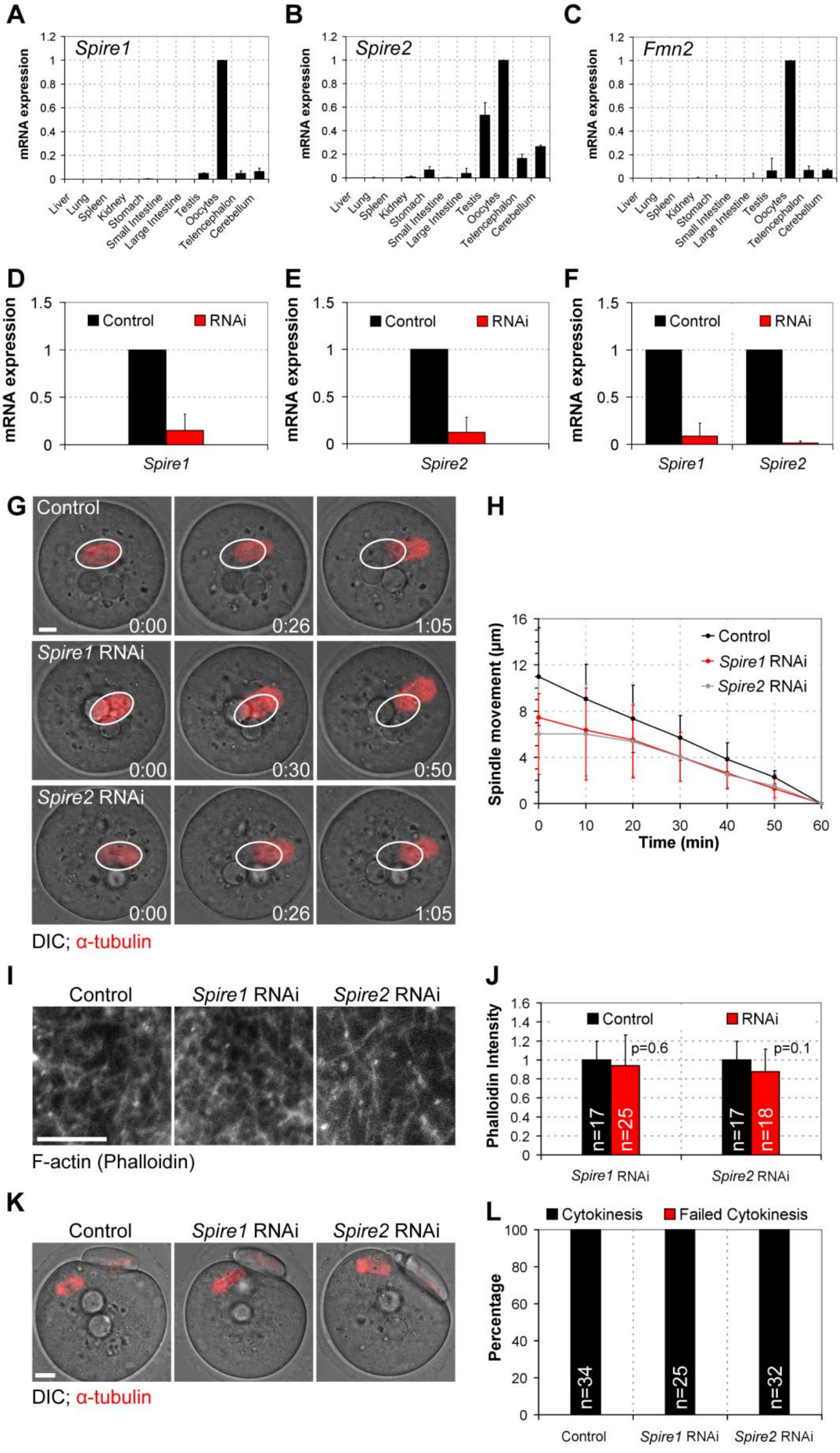

Figure S1. Individual depletion of *Spire1* or *Spire2* does not block asymmetric spindle positioning, actin network formation or polar body extrusion

(A-C) The relative expression of *Spire1* (A), *Spire2* (B) and *Fmn2* (C) in different tissues and oocytes was measured by quantitative real-time PCR. mRNA levels were normalized to those in oocytes. Data are mean±s.d. from two independent experiments.

(D) mRNA levels of *Spire1* were determined by quantitative real-time PCR in oocytes injected with scrambled negative control siRNA (Control) and *Spire1* siRNAs (RNAi). mRNA levels were normalized to those in oocytes injected with scrambled negative control siRNA. Data are mean±SEM from two independent experiments.

(E) mRNA levels of *Spire2* were determined by quantitative real-time PCR in oocytes injected with scrambled negative control siRNA (Control) and *Spire2* siRNAs (RNAi). mRNA levels were normalized to those in oocytes injected with scrambled negative control siRNA. Data are mean±SEM from two independent experiments.

(F) mRNA levels of *Spire1* and *Spire2* were determined by quantitative real-time PCR in oocytes injected with scrambled negative control siRNA (Control) and *Spire1* and *Spire2* siRNAs. mRNA levels were normalized to those in oocytes injected with scrambled negative control siRNA. Data are mean±SEM from three independent experiments.

(G) Spindle movements in live oocytes expressing mCherry-α-tubulin (red, microtubules, merged with DIC). Oocytes were injected with scrambled negative control siRNA (Control), *Spire1* siRNAs (*Spire1* RNAi) and *Spire2* siRNAs (*Spire2* RNAi). White ovals mark initial spindle positions. Time: hr:min. Scale bar: 10 µm.

(H) The spindle was tracked in oocytes in 3D data sets (13 sections, every 7 µm) as shown in (G). Spindle movements in control (black; v=0.17±0.05 µm/min; n=10), *Spire1* RNAi (red; v=0.13±0.05 µm/min; n=10; p=0.07) and *Spire2* RNAi (grey; v=0.13±0.08 µm/min; n=12; p=0.2)

(I) Oocytes from control, *Spire1* RNAi and *Spire2* RNAi were fixed during asymmetric spindle positioning (4-5 hours after germinal vesicle breakdown) and stained with Alexa Fluor 488 phalloidin to label F-actin. Scale bar: 5 µm.

(J) The mean intensity of the cytoplasmic phalloidin staining was measured in oocytes as shown in (I) that were injected with control siRNA (Control; black), with *Spire1* siRNAs (*Spire1* RNAi; red) and *Spire2* siRNAs (*Spire2* RNAi; red). Data are mean±s.d.. P-values were calculated with Student's t-test.

(K) Polar body extrusion in oocytes that were injected with scrambled negative control siRNA (Control), with *Spire1* siRNAs (*Spire1* RNAi) or *Spire2* siRNAs (*Spire2* RNAi). Scale bar: 10 µm.

(L) Polar body extrusion efficiency was scored for oocytes that progressed into anaphase and were injected with scrambled negative control siRNA (Control), *Spire1* siRNAs (*Spire1* RNAi) and *Spire2* siRNAs (*Spire2* RNAi).

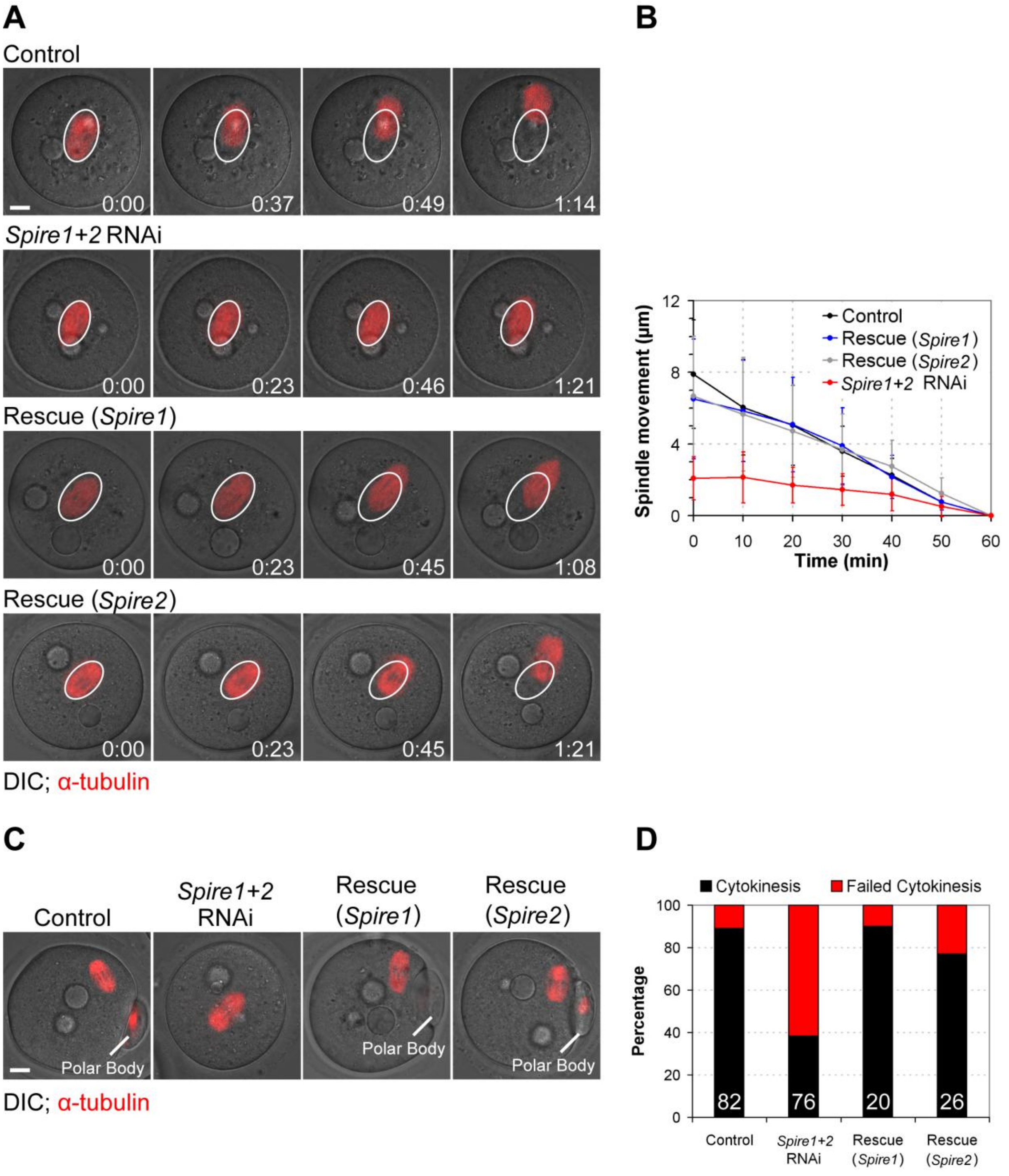

Figure S2. Individual overexpression of human *Spire1* or *Spire2* rescues asymmetric spindle positioning and polar body extrusion

(A) Spindle movements in live oocytes expressing EGFP-α-tubulin or mCherry-α-tubulin (red, microtubules, merged with DIC). Oocytes were injected with scrambled negative control siRNA (Control), *Spire1* and *Spire2* siRNAs (*Spire1+2* RNAi) and *Spire1* and *Spire2* siRNAs together with human *Spire1* (Rescue (*Spire1*)) or *Spire2* mRNAs (Rescue (*Spire2*)). White ovals mark initial spindle positions. Time: hr:min. Scale bar: 10 µm.

(B) The spindle was tracked in oocytes in 3D data sets (13 sections, every 7 µm) as shown in (A). Spindle movements in control (black; v=0.12±0.05 µm/min; n=17), *Spire1+2* RNAi (red; v=0.04±0.03 µm/min; n=13; $p \leq 10^{-5}$) and rescue experiments with human *Spire1* (blue; v=0.11±0.06 µm/min; n=7; p=0.6) or with human *Spire2* (grey; v=0.12±0.06 µm/min; n=7; p=0.94).

(C) Representative metaphase II oocytes expressing EGFP-α-tubulin or mCherry-α-tubulin (red, microtubules, merged with DIC) that were injected with scrambled negative control siRNA (Control), with *Spire1+2* siRNAs (*Spire1+2* RNAi) and *Spire1+2* siRNAs together with human *Spire1* (Rescue (*Spire1*)) or human *Spire2* mRNA (Rescue (*Spire2*)). Scale bar: 10 µm.

(D) Polar body extrusion efficiency was scored for oocytes that progressed into anaphase and were injected with scrambled negative control siRNA (Control), *Spire1+2* siRNAs (*Spire1+2* RNAi), *Spire1+2* siRNAs together with human *Spire1* (Rescue (*Spire1*)) or human *Spire2* mRNAs (Rescue (*Spire2*)). Oocyte numbers are indicated in white on the columns.

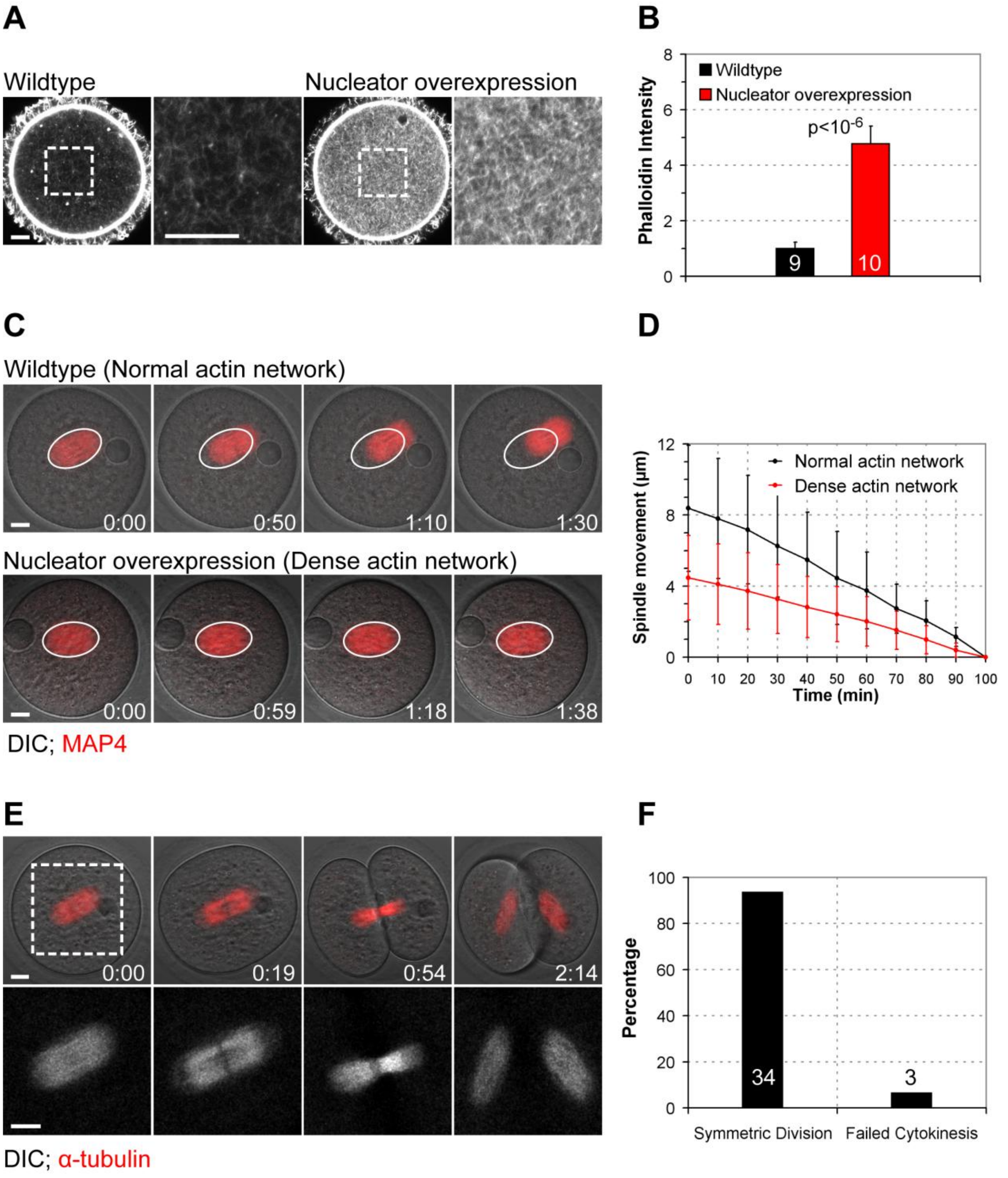

Figure S3. Mouse oocytes divide symmetrically if asymmetric spindle positioning is blocked by artificially increasing the network density

(A) Wildtype oocytes (Wildtype) and oocytes highly overexpressing *Spire1+2* and *Fmn2* were fixed during asymmetric spindle positioning and stained with Alexa Fluor 488 phalloidin to label F-actin. Boxed regions are magnified in the right panel. Scale bars: 10 µm

(B) The mean intensity of the actin network was measured in oocytes as shown in (A) that were wildtype (black column) or overexpressing *Spire1+2* and *Fmn2* (red column). Data are means±s.d. P-values were calculated with Student's t-test.

(C) Spindle movements in live oocytes expressing EGFP-MAP4 (red, microtubules, merged with DIC). Oocytes were wildtype (Wildtype) or overexpressing *Spire1+2* and *Fmn2* (Nucleator overexpression). White ovals mark initial spindle positions. Time: hr:min. Scale bar: 10 µm.

(D) The spindle was tracked in wildtype oocytes (Wildtype; black; v=0.08±0.03 µm/min; n=19) and oocytes overexpressing *Spire1+2* and *Fmn2* (Nucleator overexpression; red; v=0.05±0.02 µm/min; n=14; $p \leq 10^{-3}$; time points interpolated to 10 min intervals) in 3D data sets (13 sections, every 7 µm) as shown in (C).

(E) Anaphase in live oocytes expressing EGFP-α-tubulin (red, microtubules) merged with DIC. Asymmetric spindle positioning was blocked by severely overexpressing *Spire1* and *Spire2*. Boxed regions are magnified without DIC below. Scale bars: 10 µm. Time: hr:min.

(F) Live oocytes were labelled with EGFP-α-tubulin and asymmetric spindle positioning was blocked by severely overexpressing *Spire1+2/Fmn2*. Oocytes were monitored by long term time-lapse microscopy and symmetric division or cytokinetic failure upon anaphase onset were scored.

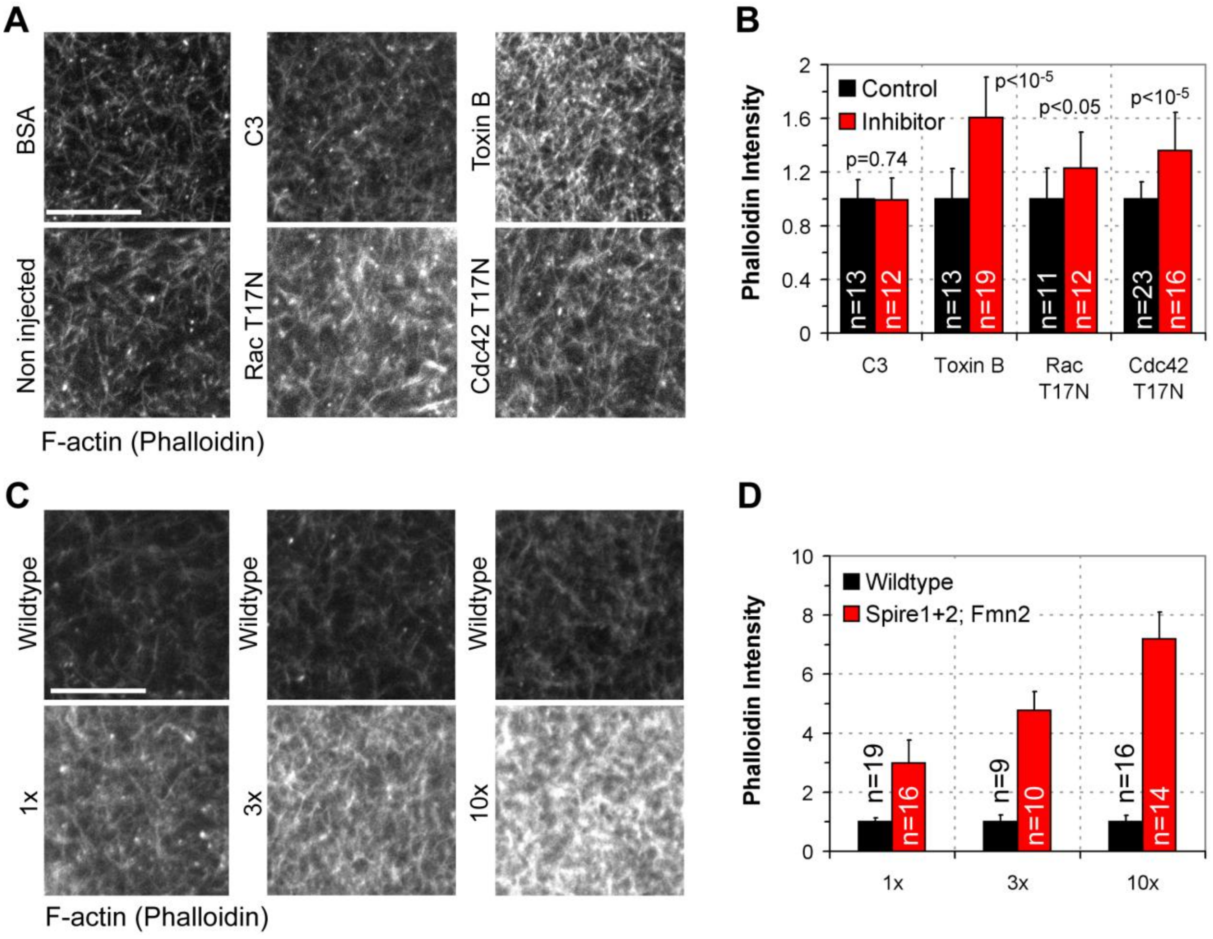

Figure S4. Network assembly is not dependent on Rho GTPase activity, but is controlled by the expression level of *Spire1/Spire2* and *Fmn2*

(A) Oocytes that were injected with 10 pl of 60 µg/ml BSA, 60 µg/ml Exoenzyme C3 (final c in oocyte: 120 nM), exposed to 3 µg/ml Toxin B or injected with mRNA encoding Rac1T17N or Cdc42T17N were fixed during asymmetric spindle positioning and stained with Alexa Fluor 488 phalloidin to label F-actin. Different conditions are specified on the left side of the images. Scale bars: 10 µm.

(B) The mean intensity of the cytoplasmic phalloidin staining was measured in oocytes as shown in (A) that were injected with 10 pl of 60 µg/ml BSA, 60 µg/ml Exoenzyme C3 (final c in oocyte: 120 nM), exposed to 3 µg/ml Toxin B or injected with mRNA encoding Rac1T17N or Cdc42T17N. Data are mean±s.d.. P-values were calculated with Student's t-test. Quantification of the density of the actin network revealed that Rho GTPases are not required to activate Spire1/Spire2 and Fmn2 dependent actin nucleation in mouse oocytes. Instead, inhibition of Cdc42 and Rac1 and treatment with Toxin B slightly increased the density of the actin network. This could be due to a direct inhibitory effect of Rho GTPases

on Spire1/Spire2 and Fmn2 dependent F-actin nucleation, or due to indirect effects because more monomeric actin is available for Spire1/Spire2 and Fmn2 when other actin nucleators that are Cdc42 and Rac1 dependent are inactive.

(C) Wildtype oocytes (Wildtype) and oocytes injected with different concentrations of mRNAs encoding Spire1-mCherry, Spire2-mCherry and Fmn2-mCherry (lower row; 1x, 3x and 10x) were fixed during asymmetric spindle positioning and stained with Alexa Fluor 488 phalloidin to label F-actin.

(D) The mean cytoplasmic phalloidin staining was measured in oocytes as shown in (C) that were injected with different concentrations of mRNAs encoding Spire1-mCherry, Spire2-mCherry and Fmn2-mCherry (red columns; 1x, 3x and 10x) and compared to the intensity in wildtype oocytes (black columns; 1x, 3x and 10x). Data are means±s.d. P-values were calculated with Student's t-test.

Supplemental Movies

Supplemental Experimental Procedures

Measurement of the cytoplasmic network density

The quantify the density of the cytoplasmic actin network, we measured the mean intensity of Alexa Fluor 488 phalloidin staining in the cytoplasm and in a region outside of the oocyte for background subtraction. Images in control and perturbed situations were acquired with identical imaging conditions and care was taken that images were not saturated during acquisition. To accurately measure comparable intensities in different oocytes, images for quantification were always acquired in the centre of the oocyte as determined by the maximum radius of the oocyte. Average, standard deviation, and statistical significance based on Student's t-test were calculated in Excel.

Automated 3D tracking of the spindle

To measure the kinetics of asymmetric spindle positioning, we injected oocytes with mRNA encoding fluorescently labelled α-tubulin or MAP4 to label microtubules. We then recorded z-stacks of the entire oocyte volume during asymmetric spindle positioning using Zeiss' MultiTime Series macro. We corrected for drifts during image acquisition with the 'correct drift' function of Imaris (Bitplane) after segmenting the

oocyte volume by low thresholding on the soluble pool of the fluorescent reporter. Afterwards we segmented the spindle by applying a higher threshold and tracked the spindle's centre of mass during asymmetric positioning using Imaris.

For averaging of asymmetric spindle positioning in different oocytes, we temporally aligned the different data sets to the time when the spindle slowed down due to arrival at the cortex. In Spire1 and Spire2 co-depleted oocytes or oocytes from Formin-2 knockout mice, where the spindle did not reach the cortex, the data sets were aligned to time points corresponding to the end of asymmetric spindle positioning in controls. The distance of the spindle to the alignment position was calculated for each time point by processing the spindle coordinates in Excel and plotted over time. To calculate average velocities of asymmetric spindle positioning, the spindle velocity in each oocyte was calculated by linear regression analysis of the displacement plots. Average, standard deviation, and statistical significance based on Student's t-test were calculated in Excel.

qRT-PCR

For qRT-PCR results in Figures S1A-S1C, mRNA was extracted from tissues using TRIzol reagent (Invitrogen) and cDNA was generated using the QuantiTec Reverse Transcription Kit (Qiagen). Real-time PCR was performed with the Light Cycler 480 (Roche) using SYBR Green. *β-microglobulin* mRNA was used for normalization. The following primer sets were used: *Spire1*, forward, 5´ agc tct gct tct gtt gcc ga 3´, reverse, 5´ ctc gaa cag ctt tcc ccc c 3´; *Spire2*, forward, 5´ aaa tca agc agg agc gga gg 3´, reverse 5´ ggt ggg ggc ttt gag cag ga 3´ (194 bp fragment); *Fmn1*, forward, 5´ agc tgg tgt gta agg agt cc 3´, reverse, 5´ gct ggg ggt gac ctc ctt ct 3´ (194 bp fragment); *Fmn2*, forward, 5´ gtg agg cgg aag ccg gta aa 3´, reverse, 5´ aca cct cct

tct cgc cga gt 3´ (201 bp fragment); *β-microglobulin*, forward, 5´ atg gga agc cga aca tact g 3´, reverse, 5´ cag tct cag tgg ggg tga at 3´.

For qRT-PCR results in Figure S1D-S1F, mRNA was extracted using an RNeasy Mini Kit (Qiagen) and cDNA was generated using the High Capacity RNA-to-cDNA Kit from (Applied Biosystems). Real-time PCR was performed with the 7900 HT Real-Time Fast PCR System (Applied Biosystems) using SYBR Green. *GAPDH* mRNA was used for normalization. The following primer sets were used: *Spire1*, forward, 5' gacagcctctgactctgaggagg 3'; *Spire1*, reverse, 5' gggcaagaattttggaggcttcttc 3'; *Spire2*, forward, taccaccagccagctcagaag 3', *Spire2*, reverse, 5' tcgatgagcctttccagctgc 3'; *GAPDH*, forward, 5' agagctgaacgggaagctcact 3'; *GAPDH*, reverse, 5' tgcctgcttcaccaccttcttgat 3'.

siRNA sequences

The following siRNAs from Qiagen were used:

Mm-Spire1_1 SI01431591 AAGGTAGAAAGTATAGAAATA
Mm-Spire1_2 SI01431598 TGCATTGAAATTTATAGTTTA
Mm_Spire1_3 SI01421605 CACCATCATTAAGATACAGTA
Mm-Spire1_4 SI01431612 TTCGGATAATTTCCTGACTAA

Mm_Spire2_1 SI01431619 CAGGAACTATAAGCTGCGCAA
Mm_Spire2_2 SI01431626 CAGAGGGTGCCAACTACCAAA
Mm_Spire2_3 SI01431633 ACCCTTGCGCATGTACATATA
Mm_Spire2_4 SI01431640 TTCCTGTAGCGTAAAGATGAA

For the data displayed in Figure 1F, the following siRNA sets were used:

Oligos A: Mm_Spire1_1, Mm_Spire1_3, Mm_Spire2_1, Mm_Spire2_4
Oligos B: Mm_Spire1_2, Mm_Spire1_4, Mm_Spire2_2, Mm_Spire2_3
Oligos C: Mm_Spire1_1, Mm_Spire2_4

Oligos A were used for all other co-depletion experiments and all rescue experiments. For individual depletion of *Spire1* and *Spire2* in Figures S1 and S2, siRNAs corresponding to Oligos A (Mm_Spire1_1, Mm_Spire1_3 and Mm_Spire2_1, Mm_Spire2_4) were used.

All control oocytes in RNAi experiments were injected with scrambled negative control siRNA.
