# Supplementary figures and images for: Spire-Type Actin Nucleators Cooperate with Formin-2 to Drive Asymmetric Oocyte Division
Source: Curr Biol. 2011 Jun 7;21(11):955–60. doi: 10.1016/j.cub.2011.04.029 (PMC3128265; doi:10.1016/j.cub.2011.04.029)

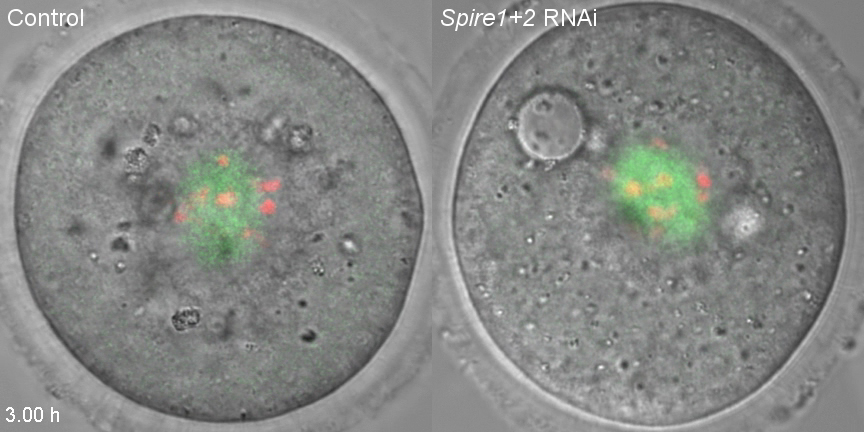

Supplement: Movie S1. Spire1 and Spire2 Drive Asymmetric Spindle Positioning. Related to Figure 1 — Time-lapse imaging (one image every 10 min) during asymmetric spindle positioning of oocytes expressing mEGFP-α-tubulin (green, microtubules) and H2B-mRFP (red, chromosomes). Oocytes were injected with scrambled negative control siRNA (left) and Spire1 and Spire2 siRNAs (right). [file mmc2.jpg]

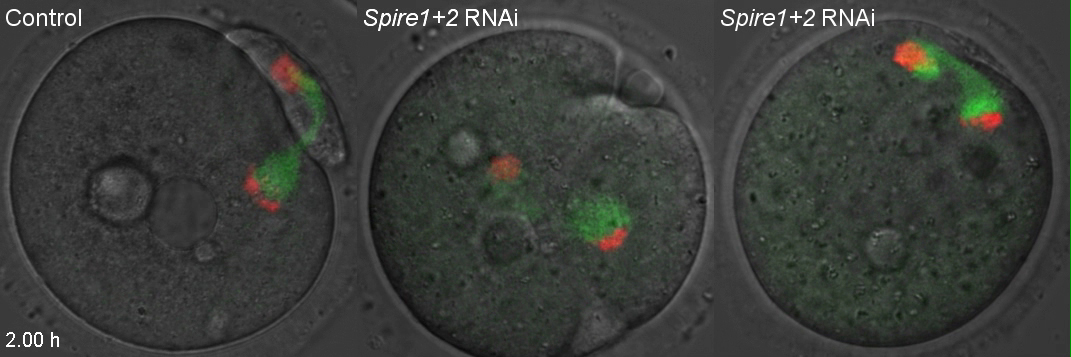

Supplement: Movie S2. Spire1 and Spire2 Drive Polar Body Extrusion. Related to Figure 2 — Time-lapse imaging (one image every 10 min) during anaphase of oocytes expressing mEGFP-α-tubulin (green, microtubules) and H2B-mRFP (red, chromosomes). Oocytes were injected with scrambled negative control siRNA (left) and Spire1 and Spire2 siRNAs (middle and right). Same oocytes as in Figure 2D. [file mmc3.jpg]

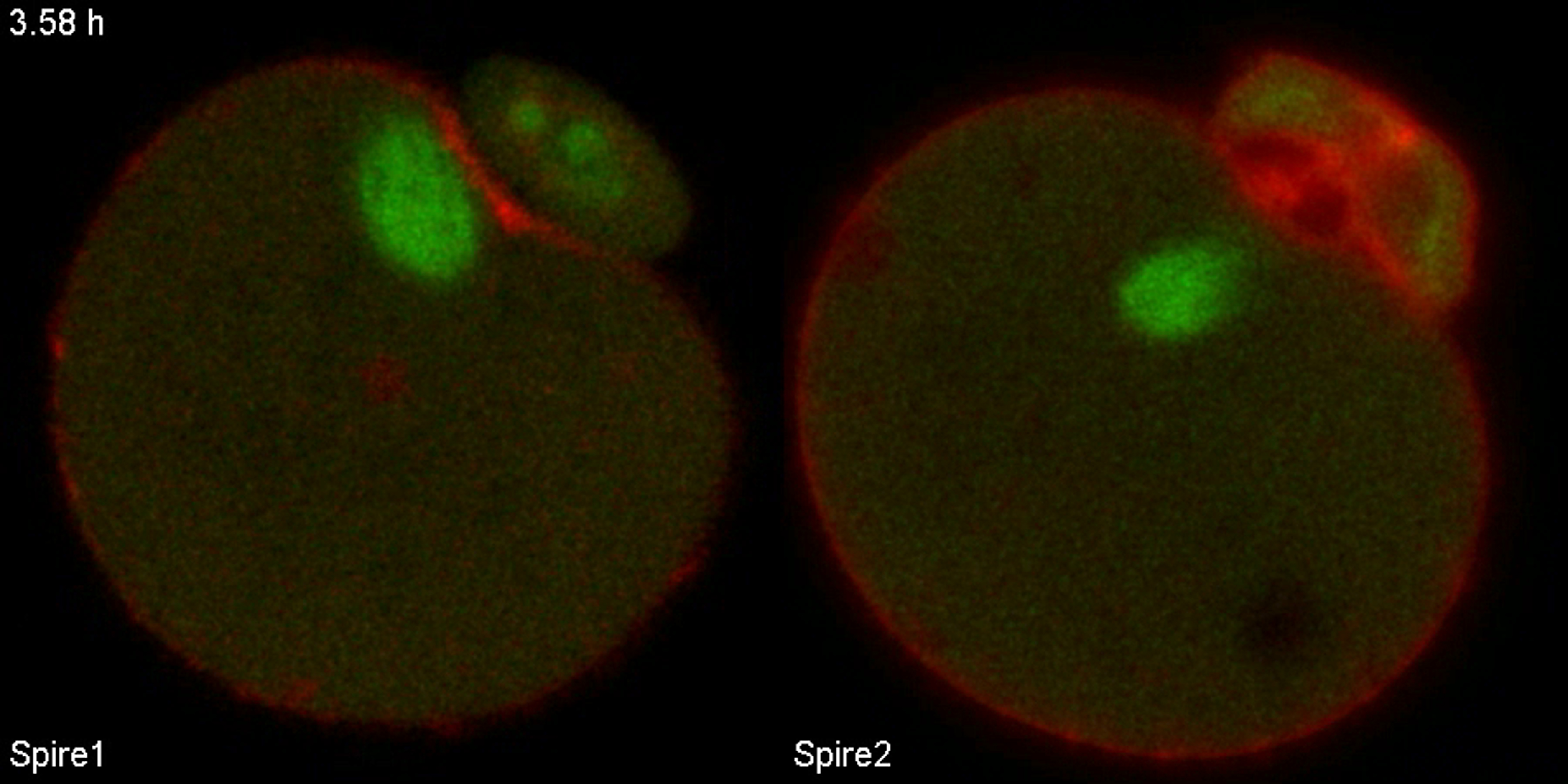

Supplement: Movie S3. Spire1 and Spire2 Localize to the Cleavage Furrow during Polar Body Extrusion. Related to Figure 2 — Time-lapse imaging (one image every 5 min) during anaphase in ooocytes overexpressing mEGFP−α-tubulin (green, microtubules) and Spire1-mCherry (red; left) or Spire2-mCherry (red; right). Same oocytes as in Figure 2E. [file mmc4.jpg]
